# Repetitive transcranial magnetic stimulation for Alzheimer’s disease: an overview of systematic reviews and meta-analysis

**DOI:** 10.3389/fnagi.2024.1383278

**Published:** 2024-03-20

**Authors:** Hua Xue, Ya-xin Li, Ya-song Xiao, Wen-hui Fan, Hong-xian He

**Affiliations:** ^1^Department of Neurology, Sichuan Taikang Hospital, Chengdu, Sichuan, China; ^2^Department of Geriatric, Sichuan Taikang Hospital, Chengdu, Sichuan, China; ^3^Department of Rehabilitation, Sichuan Taikang Hospital, Chengdu, Sichuan, China

**Keywords:** repetitive transcranial magnetic stimulation, Alzheimer’s disease, overview, meta-analysis, noninvasive brain stimulation

## Abstract

**Objective:**

Alzheimer’s disease (AD) is a prevalent neurodegenerative condition that significantly impacts both individuals and society. This study aims to evaluate the effectiveness of repetitive transcranial magnetic stimulation (rTMS) as a treatment for AD by summarizing the evidence from systematic reviews (SRs) and meta-analyses (MAs).

**Methods:**

SRs/MAs of rTMS for AD were collected by searching Embase, Web of Science, Cochrane Library, PubMed, CNKI, VIP, Sino-Med, and Wanfang databases. The search was conducted from database creation to January 23, 2024. Methodological quality, reporting quality and risk of bias were assessed using the Assessing Methodological Quality of Systematic Reviews 2 (AMSTAR-2), Risk of Bias in Systematic Reviews (ROBIS) tool and the Preferred Reporting Items for Systematic Reviews and Meta-Analyses (PRISMA). In addition, the quality of evidence for outcome measures was assessed using the Grading of Recommendations, Assessment, Development and Evaluation (GRADE).

**Results:**

Eight SRs/MAs included in this study met the inclusion criteria. Based on the AMSTAR-2, 4 of the SRs/MA were classified as low quality, while the remaining 4 were deemed to be of very low quality. The PRISMA analysis revealed that out of the 27 items reporting, 16 achieved full reporting (100%). However, there were still some deficiencies in reporting, particularly related to protocol and registration, search strategy, risk of bias, and additional analysis. The ROBIS tool indicated that only 3 SRs/MAs had a low risk of bias. The GRADE assessment indicated that 6 outcomes were of moderate quality (18.75%), 16 were of low quality (50%), and 10 were classified as very low quality (31.25%).

**Conclusion:**

Based on the evidence collected, rTMS appears to be effective in improving cognitive function in AD patients, although the methodological quality of the SRs/MAs reduces the reliability of the conclusions and the overall quality is low. However, based on the available results, we still support the value of rTMS as an intervention to improve cognitive function in AD. In future studies, it is necessary to confirm the efficacy of rTMS in AD patients and provide more reliable and scientific data to contribute to evidence-based medicine.

## 1 Introduction

Alzheimer’s disease (AD) is a neurodegenerative disease characterized by cognitive impairment, along with mental and behavioral abnormalities, and a decline in the ability to perform daily activities. It is the most common type of dementia and imposes a significant burden on the healthcare industry due to its high morbidity, disability, and mortality rates (Kaur et al., 2019; XXX, 2023). In 2015, approximately 47 million people worldwide suffered from dementia. It is estimated that the number of AD patients worldwide will reach 75 million by 2030, and this number is expected to increase to 131 million by 2050 (Watermeyer and Calia, 2019). Currently, the pathogenesis of AD is not well understood, and there are no definitive treatment options. However, the main recognized pathological features include neuroinflammatory plaques containing amyloid-β (Aβ), neural fibrillary tangles (NFTs) formed by aberrantly phosphorylated aggregates of tau proteins, and neuroinflammation (Braak et al., 2006; Silva et al., 2019; van der Kant et al., 2020; Leng and Edison, 2021). Carboplatin, donepezil, galantamine, etc. are conventional pharmacologic treatments for AD, and their mechanism of action is to inhibit acetylcholinesterase, which increases acetylcholine (Ach) levels in the brain (Birks and Harvey, 2018; Marucci et al., 2021; Pardo-Moreno et al., 2022). As a non-competitive N-methyl-D-aspartate (NMDA) receptor antagonist, memantine has been approved by the FDA for the treatment of AD, which can block the abnormal elevation of glutamate to damage neurons and reduce apoptosis of neuronal memory cells, and is mainly used in patients with moderate to severe AD (Wang and Reddy, 2017). Although these drugs provide some relief, they do not prevent disease-related neuropathological changes. Consequently, an increasing number of scientists and clinicians have begun to explore non-pharmacological treatments.

Transcranial magnetic stimulation (TMS) is a magnetic field stimulation technology that utilizes pulsed magnetic fields to act on the central nervous system (Barker et al., 1985). This process changes the membrane potential of cortical nerve cells, resulting in the generation of induced currents. These currents then affect brain metabolism and neural electrical activity, leading to a series of physiological and biochemical reactions (Kim et al., 2019; Begemann et al., 2020; Jannati et al., 2023). Repetitive TMS (rTMS), a mode of TMS, involves applying repeated magnetic signals to the cortex. By generating the sum of excitatory postsynaptic potentials, it extends the stimulation period and regulates the activity of brain areas associated with cognitive functions, thereby improving cognitive knowledge (Nguyen et al., 2018; Bao et al., 2021). Different stimulation sites of rTMS target various cognitive areas. Currently, rTMS is the most commonly used method, capable of altering cortical inhibition and excitation based on the stimulation frequency. Notably, rTMS has significant effects on cognitive enhancement. Varying frequencies of rTMS produce different regulatory effects on the cortex (Lefaucheur et al., 2020). High-frequency rTMS (>1 Hz) typically exhibits an excitatory effect, while low-frequency rTMS (≤1 Hz) often displays an inhibitory effect. Furthermore, the excitatory or inhibitory effects of rTMS may be reversed as the duration of the stimulation train changes (O’Reardon et al., 2007). The efficacy of rTMS is dependent on precise targeting to ensure the appropriate brain regions are being treated. It is essential for the physician to manually hold the stimulation coil or use a fixed bracket to secure it in place. Due to the inability to accurately sense the fit and angle of each stimulation coil on the scalp, subsequent treatments cannot be reliably replicated from previous sessions, leading to challenges in addressing target shift during treatment.

Systematic reviews (SRs)/Meta-analyses (MAs) are important tools for guiding evidence-based clinical practice and have been widely used in various medical disciplines in recent years. With the development of evidence-based medicine, more and more SRs/MAs have been conducted to investigate the effectiveness and safety of rTMS for AD. Overview of SRs/MAs is a widely used method for assessing the quality of evidence in evidence-based medicine. It involves collecting information on SRs/MAs that pertain to the same disease or health problem, providing a comprehensive approach to reassessing their quality. While SRs/MAs have always been considered a crucial source of reliable information, there are various factors in the evaluation process that can diminish their quality. These include incomplete literature sources, insufficient evaluation methods, and publication bias. By integrating the evidence from multiple SRs/MAs, an overview of SRs/MAs offers a more comprehensive and informative approach, thereby providing higher-quality evidence for clinical practice.

This overview utilized several assessment tools including the A Measurement Tool to Assess Systematic Reviews 2 (AMSTAR-2), Risk of Bias in Systematic Reviews (ROBIS), Preferred Reporting Item for Systematic Review and Meta analysis (PRISMA), and the Grading of Recommendations, Assessments, Developments, and Evaluations (GRADE) (Schünemann et al., 2008; Whiting et al., 2016; Shea et al., 2017; Page et al., 2021). The aim of this overview was to thoroughly evaluate the methodological quality, reporting quality, risk of bias, and evidence quality of systematic reviews and meta-analyses (SRs/MAs) on the use of rTMS in the treatment of AD. The objective was to provide a critical assessment of the quality of relevant SRs/MAs and to objectively and comprehensively evaluate the effectiveness and safety of rTMS for AD.

## 2 Methods

### 2.1 Inclusion and exclusion criteria

We included SRs/MAs based on RCTs of rTMS treatment for patients with AD, regardless of study countries, limited to Chinese and English. The patients met the diagnostic criteria of AD and were not limited by gender, age, race and course of disease. The intervention group was treated with rTMS without limitation of frequency, treatment site, and other treatment parameters, or rTMS in combination with other treatments, including cognitive training, acupuncture, rehabilitation therapy, and conventional medication for AD. The control group was treated with conventional medication for AD, cognitive training, rehabilitation therapy, sham stimulation, or with a blank control, which should be consistent with the treatment group at baseline. At least one of these results had to be reported in SRs/MAs: Alzheimer’s disease assessment cognitive scale (ADAS-cog), Mini-Mental State Examination (MMSE), mood, or behavior.

We excluded SRs/MAs if it met any of the following criteria: (a) duplicate published literature; (b) literature comparisons were made between two rTMS methods; (c) literature for which data could not be extracted or full text was not available; (d) other types of research, such as protocols, network meta-analyses, conference papers, and case reports.

### 2.2 Search strategy

We conducted a comprehensive search of SRs/MAs for rTMS treatment of AD from seven databases, including Embase, Web of Science, Cochrane Library, PubMed, China National Knowledge Infrastructure (CNKI), VIP, Sino-Med, and Wanfang databases published from the beginning to January 23, 2024. Search terms included: “Alzheimer’s disease,” “repetitive transcranial magnetic stimulation,” “transcranial magnetic stimulation,” “meta-analysis,” “systematic review.” We use Boolean logic to formulate search formulas that work across all databases. Detailed search strategy is provided in the Supplementary material.

### 2.3 Study selection and data extraction

According to the comprehensive search strategy, the SRs/MAs obtained from the search was imported into Endnote to remove duplicate articles. Two reviewers removed irrelevant articles and selected articles of interest by reading titles and abstracts. Two reviewers made the final decision based on the inclusion criteria by reading the full text. In case of disagreement, a third reviewer was consulted. Data were extracted from the included literature, and information extracted included: authors, country, age of patients, type of study, number of documents, sample size, treatment group, control group, methodological evaluation tools, outcome indicators, and main conclusions.

### 2.4 Assessment methods

#### 2.4.1 Methodological quality assessment

Two reviewers utilized the AMSTAR-2 tool to assess the methodological quality of the included systematic reviews. The AMSTAR-2 comprises a total of 16 items, each categorized as “Yes,” “No,” or “Partially Yes.” Items 2, 4, 7, 9, 11, 13, and 15 are considered critical (Shea et al., 2017). If there are no items defects or only one non-critical items defect, the method is deemed of high quality, resulting in an accurate and comprehensive SRs/MAs conclusion. If there are multiple non-critical items defects but no critical items defect, the method quality is considered moderate, and the SRs/MAs conclusion remains accurate. If there is a critical entry defect, regardless of the presence or absence of non-critical entry defects, the method quality is classified as low, and the SRs/MAs conclusion is also low. If there are multiple critical items defects, with or without non-critical items defects, the method quality is critical low.

#### 2.4.2 Report quality assessment

The reporting quality of the included SRs/MAs was evaluated by two independent reviewers using the PRISMA 2020 checklist (Page et al., 2021). The PRISMA statement consists of 27 items in seven fields, each of which is scored according to the degree of conformity with the literature. If the criterion is met, it is rated as “Yes,” if it is partially met, it is rated as “Partially Yes,” if it is not mentioned, it is rated as “No.”

#### 2.4.3 Risk of bias

The risk of bias of the included SR/MAs was assessed using the Risk of Bias in Systematic Reviews (ROBIS) tool (Whiting et al., 2016). The ROBIS tool assessment consisted of three phases, assessing the relevance, the extent of risk of bias (ROB) in the SRs/MAs process, and judging ROB. The second phase systematically assessed four key areas, including (a) study eligibility criteria, (b) identification and selection of studies, (c) data collection and study evaluation, and (d) synthesis and conclusions. The assessments were categorized as “high risk,” “low risk” or “unknown risk.”

#### 2.4.4 Evidence quality assessment

Two reviewers independently used the Recommended Assessment, Development, and Evaluation (GRADE) tool to assess the quality of evidence (Schünemann et al., 2008). It includes five aspects: the limitations of the study, the inconsistency of the results, the indirectness of the evidence, the imprecision and the bias of the report. We divide the quality of evidence into four levels: “High,” “Moderate,” “Low”, and “Very low.”

## 3 Results

### 3.1 Search results

**Figure 1:**
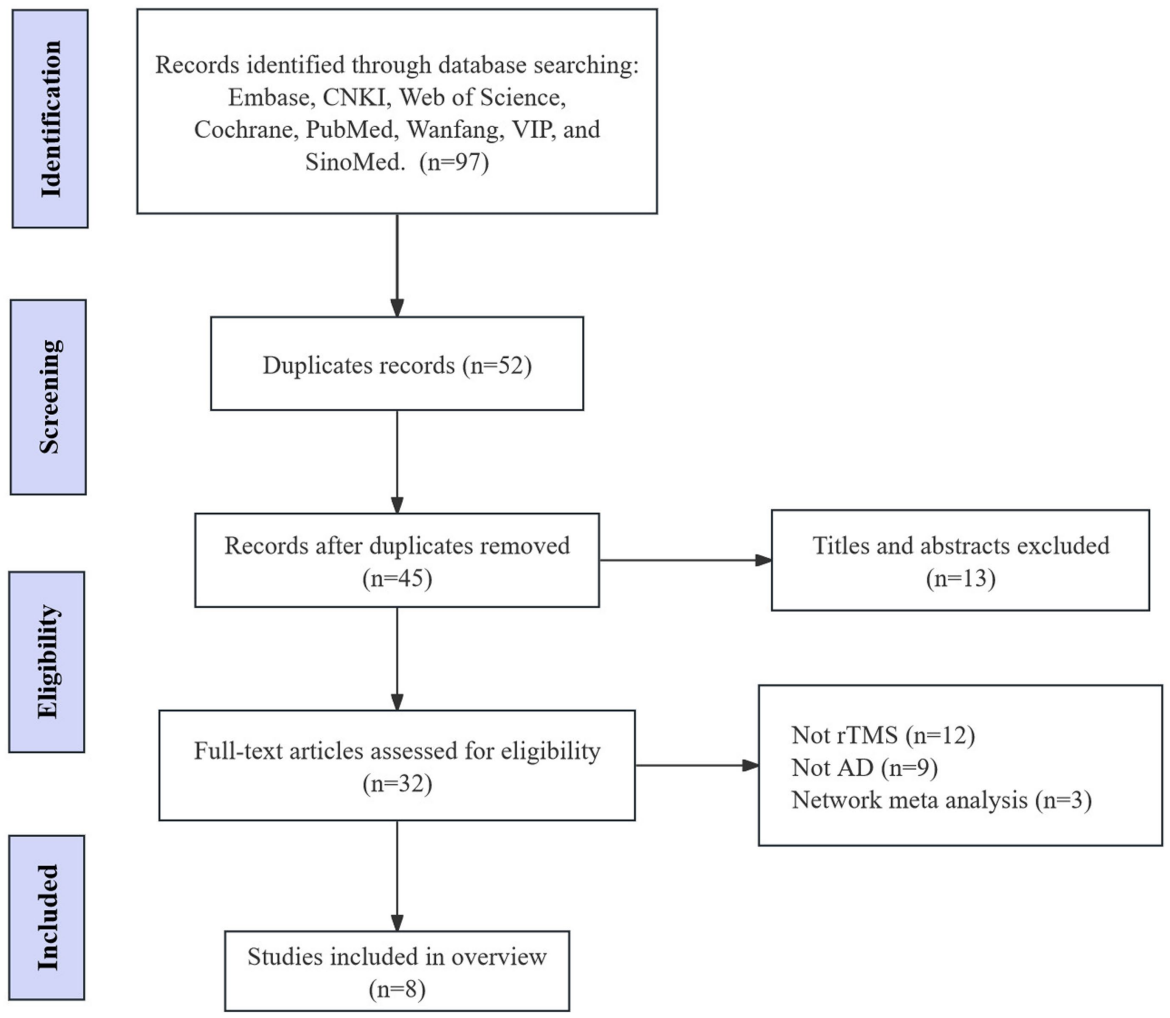
Flow chart of the literature search and study selection process.

According to the search strategy, we retrieved 97 records. Among these studies, we excluded 52 duplicate papers. Thirteen papers were excluded by reading titles and abstracts. 32 articles were considered potentially eligible and the full texts of the papers were downloaded. After independent review by two authors, 24 SRs/MAs were excluded and 8 SRs/MAs were included for final analysis (Figure 1) (Liao et al., 2015; Cheng and Wu, 2016; Liang et al., 2017; Dong et al., 2018; Lin et al., 2019; Wang et al., 2020; Zhang et al., 2022; Xiu et al., 2024).

### 3.2 Characteristics of the included SRs/MAs

The basic characteristics of the included SRs/MAs are shown in Table 1. A total of 8 SRs/MAs were included, published in studies from 2015 to 2024. Six of the SRs/MAs were published in English, and the rest were published in Chinese. The number of original studies included in each SRs/MAs ranged from 5 to 12, with a maximum sample size of 1,161 participants and a minimum sample size of 94 participants. All SRs/MAs were assessed for risk using the Cochrane risk of bias tool. Interventions in the control group were mainly conventional medication, sham stimulation, conventional therapy or combination therapy to improve cognition. The experimental group had a variety of interventions, including unilateral high-frequency rTMS treatment, unilateral low-frequency rTMS treatment, bilateral low-frequency rTMS treatment, and rTMS combined with conventional treatment. Single-site stimulation included the right and left dorsolateral prefrontal cortex (DLPFC), PC, motor cortex, inferior frontal gyrus (IFG), and superior temporal gyrus (STG), and multi-site stimulation included two or more of these sites. Outcome measures included the Picture Naming Test Score (PNTS), Neuropsychiatric Inventory (NPI), Alzheimer’s Disease Cognitive Assessment Scale (ADAS-cog), Brief Mental State Examination (MMSE), Instrumental Activities of Daily Living (IADL), Geriatric Depression Scale (GDS), and Clinician’s General Impression of Change (CGIC). Only 2 of the 8 SRs/MAs reported the duration of follow-up after treatment, which ranged from 1 to 3 months. Only 3 SRs/MAs described disease staging.

**Table 1:** Characteristics of the included systematic reviews.

Included studies	No. of RCTs	Participants	Experimental intervention	Control intervention	Basic features of rTMS	Risk assessment tools	Meta-analyses	Adverse effects	Follow up	Disease type	Outcomes	Main conclusion
Cheng and Wu (2016)	9	258	rTMS, rTMS + routine treatment	Sham stimulation, sham stimulation + routine treatment	5/10/20 HZ, left DLPFC/bilateral DLPFC	Cochrane risk of bias tool	Yes	None	Not reported	Not reported	PNTS, NPI, ADAS-cog	High-frequency rTMS has a significant improvement effect on the cognitive function of patients with AD. Correct use of stimulation therapy can achieve significant efficacy. Moreover, the therapy is painless, non-invasive, simple and easy to operate, and is an ideal method for the treatment of AD
Dong et al. (2018)	5	148	rTMS, rTMS + routine treatment	Sham stimulation, sham stimulation + routine treatment	10/20 HZ, left DLPFC/bilateral DLPFC/Broca/Wernicke/pSAC, 80–110% RMT	Cochrane risk of bias tool	Yes	Mild extrapyramidal reaction, headache	1 to 3 months	Not reported	ADAS-cog, MMSE, GDS scale, CGIC scale, IADL scale	rTMS is relatively well tolerated, with some promise for cognitive improvement and global impression inpatients with AD. Our findings also indicate the variability between ADAS-cog and MMSE in evaluating global cognitive impairment
Liang et al. (2017)	5	223	rTMS, rTMS + routine treatment	Sham stimulation, sham stimulation + routine treatment	1/10/20 HZ, left DLPFC/bilateral DLPFC	Cochrane risk of bias tool	Yes	Not reported	Not reported	Moderate/late AD	ADAS-cog, MMSE	Transcranial magnetic stimulation has significant therapeutic effect on cognitive function in AD. High frequency stimulation is significantly effective compared to the low frequency stimulation
Liao et al. (2015)	7	94	rTMS, rTMS + routine treatment	Sham stimulation, sham stimulation + routine treatment	1/10/20 HZ, left DLPFC/bilateral DLPFC/right DLPFC	Cochrane risk of bias tool	Yes	Not reported	Not reported	Mild/moderate AD	MMSE	This meta-analysis supports the notion that rTMS has a therapeutic effect on the cognition of AD patients with mild to moderate cognitive impairment. High frequency rTMS, but not low frequency rTMS, over the right DLPFC and/or bilateral DLPFC appears to be more effective in this regard
Lin et al. (2019)	12	231	rTMS, rTMS + routine treatment	Sham stimulation, sham stimulation + routine treatment	1/5/10/20 HZ, left DLPFC/bilateral DLPFC/Broca/Wernicke/pSAC/IFG/STG, 90–110% RMT, 1–20 Hz	Cochrane risk of bias tool	Yes	Not reported	Not reported	Not reported	MMSE	rTMS can significantly improve cognitive ability in patients with mild to moderate AD. Stimulation of multiple sites and long-term treatment are better at improving AD-associated cognitive performance
Wang et al. (2020)	10	240	rTMS, rTMS + routine treatment	Sham stimulation, sham stimulation + routine treatment	10/20 HZ, left DLPFC/bilateral DLPFC/Broca/Wernicke/pSAC/IFG/STG, 80–110% RMT	Cochrane risk of bias tool	Yes	Mild extrapyramidal reactions, transient headache, fatigability and painful scalp sensation	Not reported	Not reported	MMSE	rTMS was an effective therapy for cognitive impairment in AD
Xiu et al. (2024)	12	1,161	rTMS, rTMS + routine treatment	Sham stimulation, sham stimulation + routine treatment	5/10/15/20 HZ, left DLPFC/bilateral DLPFC, 80–120% RMT	Cochrane risk of bias tool	Yes	Scalp pain, loss of consciousness, cerebellar embolism, headache, tinnitus, fatigue and stimulation of regional muscle contraction	Not reported	Not reported	MMSE, MoCA, ADAS-Cog, P300	High-frequency rTMS can improve global cognitive function in elderly patients with mild to moderate AD, which is an effective and safe rehabilitation treatment tool for AD patients
Zhang et al. (2022)	9	361	rTMS, rTMS + routine treatment	Sham stimulation, sham stimulation + routine treatment	1/10/20 HZ, left DLPFC/bilateral DLPFC/Broca/Wernicke/pSAC/IFG/STG, 80–120% RMT	Cochrane risk of bias tool	Yes	Headache, fatigue	1 to 3 months	Mild/moderate AD	ADAS-cog, MMSE	rTMS is a potentially effective treatment for cognitive impairment in AD that is safe and can induce long-lasting effects

AD, Alzheimer’s disease; rTMS, repetitive transcranial magnetic stimulation; RMT, resting motor threshold; DLPFC, dorsolateral prefrontal cortex; IFG, inferior frontal gyrus; STG, superior temporal gyrus; pSAC, parietal somatosensory association cortex; PNTS, picture naming test score; NPI, neuropsychiatric inventory; ADAS-cog, Alzheimer disease assessment cognitive scale; MMSE, Mini-Mental State Examination; IADL, instrumental daily living activity; GDS, geriatric depression scale; CGIC, Clinician’s global impression of change.

### 3.3 Methodological quality assessment of the included SRs/MAs

We utilized AMSTAR-2 to assess the methodological quality of the 8 included SRs/MAs. Out of the 8 research reports, 4 were classified as critically low quality, while the remaining 4 were rated as low quality. Only two SRs/MAs received a rating of “Yes” for key item 2, while the others were rated as “No” due to the absence of a review scheme (Dong et al., 2018; Zhang et al., 2022). Regarding key item 4, only one SR/MA did not present a comprehensive and detailed search strategy (Cheng and Wu, 2016). Additionally, two SRs/MAs did not provide a list of excluded studies, resulting in a “No” rating for key item 7 (Cheng and Wu, 2016; Liang et al., 2017). Similarly, two SRs/MAs did not fully evaluate the risk of bias in individual studies, leading to a “No” rating for key item 9 (Liao et al., 2015; Liang et al., 2017). Key items 13 and 15 exhibited flaws as well. Four SRs/MAs did not elucidate the risk of bias in individual studies, and four SRs/MAs did not thoroughly investigate publication bias. Considering one or more significant flaws in key items 2, 4, 7, 9, 11, 13, and 15, as well as multiple flaws in non-key items, all SRs/MAs are deemed to be of low or critically low quality. An overview of methodological quality of included SRs/MAs is presented in Table 2.

**Table 2:** Methodological quality of included SRs/MAs.

Included studies	AMSTAR 2																Overall quality
	Q 1	Q 2*	Q 3	Q 4*	Q 5	Q 6	Q 7*	Q 8	Q 9*	Q 10	Q 11*	Q 12	Q 13*	Q 14	Q 15*	Q 16	
Cheng and Wu (2016)	Y	N	Y	N	Y	Y	N	N	Y	Y	Y	Y	Y	Y	Y	Y	CL
Dong et al. (2018)	Y	Y	Y	Y	Y	Y	Y	Y	Y	Y	Y	N	N	Y	N	Y	CL
Liang et al. (2017)	Y	N	Y	Y	Y	Y	N	Y	N	N	Y	N	N	N	N	Y	CL
Liao et al. (2015)	Y	N	Y	Y	Y	Y	Y	Y	N	Y	Y	N	N	Y	N	Y	CL
Lin et al. (2019)	Y	N	Y	Y	Y	Y	Y	Y	Y	Y	Y	Y	Y	N	Y	Y	L
Wang et al. (2020)	Y	N	Y	Y	Y	Y	Y	Y	Y	Y	Y	Y	Y	Y	Y	Y	L
Xiu et al. (2024)	Y	N	Y	Y	Y	Y	Y	Y	Y	Y	Y	Y	Y	Y	Y	Y	L
Zhang et al. (2022)	Y	Y	Y	Y	Y	Y	Y	Y	Y	Y	Y	N	N	Y	Y	Y	L
Y + PY/total (%)	100	25	100	87.5	100	100	75	87.5	75	87.5	100	50	50	75	62.5	100	

The key items of the AMSTAR 2. H: represents the ranking of quality as high; M: represents the ranking of quality as moderate; L: represents the ranking of quality as low; CL: represents the ranking of quality as critically low. Q1: did the research questions and inclusion criteria for the review include the components of PICO? Q2: did the report of the review contain an explicit statement that the review methods were established prior to the conduct of the review and did the report justify any significant deviations from the protocol? Q3: did the review authors explain their selection of the study designs for inclusion in the review? Q4: did the review authors use a comprehensive literature search strategy? Q5: did the review authors perform study selection in duplicate? Q6: did the review authors perform data extraction in duplicate? Q7: did the review authors provide a list of excluded studies and justify the exclusions? Q8: did the review authors describe the included studies in adequate detail? Q9: did the review authors use a satisfactory technique for assessing the risk of bias (RoB) in individual studies that were included in the review? Q10: did the review authors report on the sources of funding for the studies included in the review? Q11: if a meta-analysis was performed, did the review authors use appropriate methods for the statistical combination of results? Q12: if a meta-analysis was performed, did the review authors assess the potential impact of RoB in individual studies on the results of the meta-analysis or another evidence synthesis? Q13: did the review authors account for RoB in primary studies when interpreting/discussing the results of the review? Q14: did the review authors provide a satisfactory explanation for, and discussion of, any heterogeneity observed in the results of the review? Q15: if they performed quantitative synthesis did the review authors carry out an adequate investigation of publication bias (small study bias) and discuss its likely impact on the results of the review? Q16: did the review authors report any potential sources of conflict of interest, including any funding they received for conducting the review? AMSTAR 2, assessment of multiple systematic reviews 2; N, no; PY, partial yes; Y, yes.

### 3.4 Reporting quality of the included SRs/MAs

We used PRISMA to assess the reporting quality of the eight included SRs/MAs (Table 3). Among the 27 items, 16 items were fully reported (100%), and 11 items had missing or insufficient reporting. In the methods section, only 2 studies reported protocol and registration, with a reporting rate of 25% (Dong et al., 2018; Zhang et al., 2022). Limitations were found in the risk of bias in individual studies (75%), synthesis of results (87.5%), risk of bias across studies (75%), and additional analyses (87.5%). In the “Results” section, only one SRs/MAs did not describe the characteristics of the study (87.5%) (Cheng and Wu, 2016), three SRs/MAs did not describe risk of bias within studies (62.5%) (Liao et al., 2015; Liang et al., 2017; Lin et al., 2019), three SRs/MAs did not describe the risk of bias across studies (62.5%) (Liao et al., 2015; Liang et al., 2017; Lin et al., 2019), and one did not report additional analyses (87.5%) (Liang et al., 2017). Half of the SRs/MAs reported summary of the evidence, and one SRs/MAs did not mention limitations (87.5%).

**Table 3:** Results of the PRISMA checklist for the included SRs/MAs.

Section/topic	Items	Cheng and Wu (2016)	Dong et al. (2018)	Liang et al. (2017)	Liao et al. (2015)	Lin et al. (2019)	Wang et al. (2020)	Xiu et al. (2024)	Zhang et al. (2022)	Compliance (%)
Title	Q1. Title	Y	Y	Y	Y	Y	Y	Y	Y	100
Abstract	Q2. Structured summary	Y	Y	Y	Y	Y	Y	Y	Y	100
Introduction	Q3. Rationale	Y	Y	Y	Y	Y	Y	Y	Y	100
	Q4. Objectives	Y	Y	Y	Y	Y	Y	Y	Y	100
Methods	Q5. Protocol and registration	N	Y	N	N	N	N	N	Y	25
	Q6. Eligibility criteria	Y	Y	Y	Y	Y	Y	Y	Y	100
	Q7. Information sources	Y	Y	Y	Y	Y	Y	Y	Y	100
	Q8. Search	Y	Y	Y	Y	Y	Y	Y	Y	100
	Q9. Study selection	Y	Y	Y	Y	Y	Y	Y	Y	100
	Q10. Data collection process	Y	Y	Y	Y	Y	Y	Y	Y	100
	Q11. Data items	Y	Y	Y	Y	Y	Y	Y	Y	100
	Q12. Risk of bias in individual studies	Y	Y	N	N	Y	Y	Y	Y	75
	Q13. Summary measures	Y	Y	Y	Y	Y	Y	Y	Y	100
	Q14. Synthesis of results	Y	Y	N	Y	Y	Y	Y	Y	87.5
	Q15. Risk of bias across studies	Y	Y	N	N	Y	Y	Y	Y	75
	Q16. Additional analyses	Y	Y	N	Y	Y	Y	Y	Y	87.5
Results	Q17. Study selection	Y	Y	Y	Y	Y	Y	Y	Y	100
	Q18. Study characteristics	N	Y	Y	Y	Y	Y	Y	Y	87.5
	Q19. Risk of bias within studies	Y	Y	N	N	N	Y	Y	Y	62.5
	Q20. Results of individual studies	Y	Y	Y	Y	Y	Y	Y	Y	100
	Q21. Synthesis of results	Y	Y	Y	Y	Y	Y	Y	Y	100
	Q22. Risk of bias across studies	Y	Y	N	N	N	Y	Y	Y	62.5
	Q23. Additional analysis	Y	Y	N	Y	Y	Y	Y	Y	87.5
Discussion	Q24. Summary of evidence	N	Y	N	N	Y	Y	Y	N	50
	Q25. Limitations	N	Y	Y	Y	Y	Y	Y	Y	87.5
	Q26. Conclusions	Y	Y	Y	Y	Y	Y	Y	Y	100
Funding	Q27. Funding	Y	Y	Y	Y	Y	Y	Y	Y	100

N, no; PY, partial yes; Y, yes.

### 3.5 Risk of bias of the included SRs/MAs

Table 4 presents the results of the risk of bias assessment of the included SRs/MAs using the ROBIS tool. The tool consists of three stages and four areas. In the first stage, all SRs/MAs were evaluated and found to have a low risk of bias, indicating their relevance to the study topic. Domain 1 focuses on assessing the eligibility criteria of the studies, and one SRs/MAs (12.5%) was rated as having a high risk of bias (Cheng and Wu, 2016). Domain 2 involves the assessment of study identification and selection, and seven SRs/MAs (87.5%) were rated as having a low risk of bias (Cheng and Wu, 2016). Domain 3 evaluates the collection and study appraisal, and all were rated as having a low risk of bias. Domain 4 assesses the attention given to the synthesis and findings, and six SRs/MAs (75%) were rated as having a low risk of bias (Cheng and Wu, 2016; Dong et al., 2018; Lin et al., 2019; Wang et al., 2020; Zhang et al., 2022; Xiu et al., 2024). In stage 3, when considering the overall risk of bias, five SRs/MAs were rated as high risk because they did not provide any explanation or supplement regarding the risk of bias, while the remaining SRs/MAs were considered to have a low risk.

**Table 4:** Risk of bias of the included SRs/MAs.

Included studies	Phase 1	Phase 2				Phase 3
	Assessing relevance	Domain 1: study eligibility criteria	Domain 2: identification and selection of studies	Domain 3: collection and study appraisal	Domain 4: synthesis and findings	Risk of bias in the review
Cheng and Wu (2016)	low risk	high risk	high risk	low risk	low risk	low risk
Dong et al. (2018)	low risk	low risk	low risk	low risk	low risk	high risk
Liang et al. (2017)	low risk	low risk	low risk	low risk	high risk	high risk
Liao et al. (2015)	low risk	low risk	low risk	low risk	high risk	high risk
Lin et al. (2019)	low risk	low risk	low risk	low risk	low risk	high risk
Wang et al. (2020)	low risk	low risk	low risk	low risk	low risk	low risk
Xiu et al. (2024)	low risk	low risk	low risk	low risk	low risk	low risk
Zhang et al. (2022)	low risk	low risk	low risk	low risk	low risk	high risk

### 3.6 Evidence quality evaluation results

We evaluated the quality of evidence using the GRADE tool and found that 8 SRs/MAs provided evidence for 32 outcome indicators (Table 5). The results showed that there was no high-level evidence in the included outcomes, 6 were moderate evidence (18.75%), 16 were low evidence (50%), and 10 (31.25%) were very low evidence, and the quality of evidence was generally low. Overall, the quality of evidence was generally low. This could be attributed to several factors. Firstly, there might be publication bias as indicated by an asymmetric funnel plot or the presence of only positive results. Secondly, the included studies had a risk of bias due to issues such as lack of randomization, blinding, allocation concealment, incomplete outcome data, or selective reporting bias. Thirdly, the small sample size of the included studies and inconsistencies in interventions, data extraction, and outcome indicators could have affected the accuracy of the results. Lastly, there was significant heterogeneity among the studies.

**Table 5:** Results of evidence quality with GRADE.

Included studies	Outcomes	Risk of bias	Inconsistency	Indirectness	Imprecision	Publication bias	Relative effect (95% CI)	*p*-value	Quality
Cheng and Wu (2016)	PNTS (Watermeyer and Calia, 2019)	−1	0	0	−1	−1	MD 16.30 (14.03,18.57)	<0.0001	Very low
	NPI (Kaur et al., 2019)	−1	0	0	−1	−1	MD -6.42 (−8.87, −3.97)	<0.0001	Very low
	ADAS-cog (van der Kant et al., 2020)	−1	0	0	−1	−1	MD −3.63 (−4.82, −2.45)	<0.0001	Very low
Dong et al. (2018)	ADAS-cog (Watermeyer and Calia, 2019)	−1	0	0	−1	−1	MD −3.65 (−5.82, −1.48)	0.001	Very low
	MMSE (Watermeyer and Calia, 2019)	−1	0	0	0	−1	MD 0.49 (−1.45, 2.42)	0.61	Low
	GDS scale (Kaur et al., 2019)	−1	0	0	0	−1	MD −1.36 (−3.93, 1.21)	0.30	Low
	CGIC scale (Kaur et al., 2019)	−1	0	0	−1	−1	MD −0.79 (−1.24, −0.34)	0.0006	Very low
	IADL scale (Kaur et al., 2019)	−1	−1	0	−1	−1	MD 0.59 (−1.21, 2.38)	0.52	Very low
Liang et al. (2017)	MMSE (van der Kant et al., 2020)	−1	0	0	0	−1	MD 1.05 (0.21,1.88)	0.001	Low
	ADAS-cog (Watermeyer and Calia, 2019)	−1	0	0	0	−1	MD -0.71 (−1.04, −0.39)	<0.0001	Low
Liao et al. (2015)	MMSE (Silva et al., 2019)	−1	0	0	0	−1	MD 1.00 (0.41, 1.58)	0.0008	Low
	High frequency MMSE (Leng and Edison, 2021)	−1	0	0	0	−1	MD 1.06 (0.47, 1.66)	0.0004	Low
	Low frequency MMSE (Watermeyer and Calia, 2019)	−1	0	0	0	−1	MD 0.97 (−0.62, 2.50)	0.24	Low
Lin et al. (2019)	MMSE (Barker et al., 1985)	−1	0	0	0	0	MD 0.60 (0.35, 0.85)	<0.001	Moderate
	Sing site MMSE (van der Kant et al., 2020)	−1	0	0	−1	0	MD 0.13 (−0.35, 0.62)	0.60	Low
	Multiple sites MMSE (Birks and Harvey, 2018)	−1	0	0	0	0	MD 0.86 (0.18, 1.54)	0.01	Moderate
	Short term treatment MMSE (van der Kant et al., 2020)	−1	0	0	−1	−1	MD 0.29 (−1.04, 1.62)	0.67	Very low
	Long term treatment MMSE (van der Kant et al., 2020)	−1	0	0	0	−1	MD 2.77 (1.90, 2.92)	<0.0001	Very low
Wang et al. (2020)	MMSE (Pardo-Moreno et al., 2022)	−1	0	0	0	−1	MD 0.42 (0.18, 0.67)	0.0006	Moderate
	Sing site MMSE (van der Kant et al., 2020)	−1	0	0	0	0	MD 0.24 (−0.45, 0.92)	0.50	Moderate
	Multiple sites MMSE (van der Kant et al., 2020)	−1	0	0	0	0	MD 0.47 (0.14, 0.79)	0.005	Moderate
	Short term treatment MMSE (Braak et al., 2006)	−1	0	0	0	−1	MD 0.18 (−0.20, 0.56)	0.36	Low
	Long term treatment MMSE (Birks and Harvey, 2018)	−1	0	0	0	−1	MD 0.59 (0.28, 0.90)	0.0002	Low
Xiu et al. (2024)	MMSE (Pardo-Moreno et al., 2022)	−1	0	0	0	0	MD 3.64 (1.86, 5.42)	<0.0001	Moderate
	Short term treatment MMSE (Kaur et al., 2019)	−1	0	0	0	−1	MD 2.90 (−1.06, 6.86)	0.15	Low
	Long term treatment MMSE (Watermeyer and Calia, 2019)	−1	0	0	0	−1	MD 5.43 (2.84, 8.01)	<0.0001	Low
	MoCA (Silva et al., 2019)	−1	0	0	0	−1	MD 3.69 (1.84, 5.54)	<0.0001	Low
	ADAS-cog (Silva et al., 2019)	−1	0	0	0	−1	MD -3.53 (−4.91, −2.15)	<0.0001	Low
	P300 latency (Kaur et al., 2019)	−1	−1	0	0	−1	MD −38.32 (−72.40, −4.24)	0.03	Very low
	P300 amplitude (Kaur et al., 2019)	−1	−1	0	0	−1	MD 1.09 (0.45, 1.72)	<0.0001	Very low
Zhang et al. (2022)	MMSE (Braak et al., 2006)	−1	0	0	0	−1	MD 1.82 (1.41, 2.22)	<0.0001	Low
	ADAS-cog (Leng and Edison, 2021)	−1	0	0	0	−1	MD 2.72 (1.77, 3.67)	<0.0001	Low

PNTS, picture naming test score; NPI, neuropsychiatric inventory; ADAS-cog, Alzheimer disease assessment cognitive scale; MMSE, mini-mental state examination; IADL, instrumental daily living activity; GDS, geriatric depression scale; CGIC, clinician’s global impression of change.

### 3.7 Outcome measures

Most current SRs/MAs are mainly focused on the improvement of efficacy and safety of rTMS in AD patients in the following aspects: cognitive function, activities of daily living and adverse reactions.

#### 3.7.1 Improvement of cognitive symptoms

Impaired cognitive function is a common symptom of AD. These 8 SRs/MAs contained a total of 131 clinical trials. These studies selected MMSE, MoCA and ADAS-cog to assess cognitive function. Seven SRs/MAs investigated the MMSE scores of AD patients treated with rTMS (Liao et al., 2015; Liang et al., 2017; Dong et al., 2018; Lin et al., 2019; Wang et al., 2020; Zhang et al., 2022; Xiu et al., 2024). One of these 7 SRs/MAs showed that rTMS did not significantly improve the MMSE score of AD patients (MD = 0.59, 95% CI: −1.21 to 2.38, *p* = 0.52) (Dong et al., 2018), and the remaining SRs/MAs showed that rTMS could significantly improve the MMSE score of AD patients. Liao et al. further conducted a subgroup analysis based on the frequency of rTMS and found that high frequency rTMS could significantly improve the MMSE score (MD = 1.06, 95% CI: 0.47 to 1.66, *p* = 0.0004), while no significance was found for low-frequency rTMS (MD = 0.97, 95% CI: −0.62 to 2.50, *p* = 0.24) (Liao et al., 2015). Subgroup analysis of the two SRs/MAs based on stimulation site showed that multi-site stimulation improved cognition more significantly than single-site stimulation (Lin et al., 2019; Wang et al., 2020). Subgroup analysis of two SR/MA based on rTMS treatment courses showed that long-term rTMS treatment was more effective in improving cognition (Wang et al., 2020; Xiu et al., 2024).

Five SRs/MAs investigated the ADAS-cog of rTMS in the treatment of AD (Cheng and Wu, 2016; Liang et al., 2017; Dong et al., 2018; Zhang et al., 2022; Xiu et al., 2024). The pooled results of SRs/MAs showed that rTMS treatment of AD patients could significantly reduce ADAS-cog, which proved the improvement of cognitive function. Only one SRs/MAs evaluated cognition using MoCA, and this study showed that rTMS treatment of AD patients significantly improved MoCA (Xiu et al., 2024).

#### 3.7.2 Adverse reactions

Four of the included SRs/MAs reported adverse reactions after rTMS treatment. Adverse reactions include mild extrapyramidal reaction, headache, fatigability, painful scalp sensation, loss of consciousness, cerebellar embolism, tinnitus (Dong et al., 2018; Wang et al., 2020; Zhang et al., 2022; Xiu et al., 2024).

## 4 Discussion

### 4.1 Summary of main findings

This overview presents the first comprehensive descriptive analysis of SRs/MAs on the use of rTMS for AD. The analysis included 69 RCTs with a total of 2,676 participants. To assess the methodological quality, reporting quality, evidence quality, and risk of bias of the published SRs/MAs, we employed rigorous evaluation tools including AMSTAR 2, ROBIS, PRISMA, and GRADE. According to AMSTAR 2, four out of 8 SRs/MAs were rated as very low quality and 4 as low quality. Using the PRISMA checklist, we observed that out of the 27 items, 16 were fully reported while 11 were either missing or insufficiently reported. The application of the ROBIS tool indicated that 3 SRs/MAs had a low risk of bias. The GRADE results revealed that the included literature did not provide any high quality evidence. Moderate quality evidence was found in 6 outcomes (18.75%), while 16 outcomes (50%) provided low-level evidence, and 10 outcomes (31.25%) presented very low evidence. Overall, the quality of the evidence was generally low.

### 4.2 Mechanism of rTMS for Alzheimer’s disease

rTMS has been found to have an impact on the treatment of cognitive impairment, although its specific mechanism is still not fully understood. There are several possible mechanisms that have been suggested. rTMS can regulate the excitability of the cerebral cortex by altering the frequency of stimulation to specific regions of the brain (Chen et al., 2023). Different frequencies of rTMS have different effects. Low frequency stimulation (≤1 Hz) can inhibit the activity of local neurons and reduce cortical excitability, whereas high frequency stimulation (≥5 Hz) can excite the activity of local neurons and increase cortical excitability (Chen et al., 2023). High frequency stimulation can also influence the metabolism of brain functions by enhancing neural activity in the corresponding brain regions, leading to improvements in neurobiochemistry and related metabolites at the site of stimulation (Ahmed et al., 2012). This could explain why high frequency rTMS stimulation has shown better therapeutic effects in patients with AD. However, a recent controlled study demonstrated that 2 weeks of low-frequency (1 Hz) rTMS stimulation of the right DLPFC in AD patients improved recognition memory function compared to sham stimulation, and this improvement was sustained during a 1 month follow-up period (Turriziani et al., 2019). It is worth noting that rTMS stimulation has not yet been standardized in terms of frequency, and other therapeutic parameters such as high stimulation intensity may also enhance motor cortical excitability. In a meta analysis conducted by Liao et al., it was found that high-frequency rTMS (rather than low-frequency rTMS) applied to the right DLPFC and/or bilateral DLPFC appears to be more effective in improving cognition (Liao et al., 2015).

Studies have shown that patients with AD exhibit abnormal neural functional connections. rTMS has been found to regulate the synaptic plasticity and connectivity of neurons, promoting the remodeling of neurons and restoration of functional connections (Song et al., 2020). This process involves long-term potentiation (LTP) and long-term depression (LTD). High-frequency rTMS induces excitatory postsynaptic potential changes, resulting in the LTP effect. On the other hand, low-frequency rTMS induces inhibitory postsynaptic potential changes, leading to the LTD effect (Li et al., 2019). Neuroimaging-based studies have demonstrated that high-frequency rTMS increases local cortical blood perfusion and moderates cholinergic neuron damage resulting from localized blood perfusion, thereby slowing the progression of cognitive dysfunction. Dressle et al. used functional magnetic resonance imaging (fMRI) to confirm that suprathreshold rTMS increases cerebral perfusion in the cortex of the stimulation site (Dressler et al., 1990). Paus et al. used a continuous pulse sequence with a stimulation parameter of 10 s, high-frequency rTMS at a frequency of 10 Hz, and made positron emission tomography (PET) to measure the cerebral blood flow index, and found that the number of rTMS pulses was positively correlated with the cerebral blood flow index of the stimulation site (Aceves-Serrano et al., 2022). These mechanisms contribute to the therapeutic effects of rTMS in treating AD and improving cognitive function in patients. The most common adverse reactions of rTMS treatment are scalp pain during treatment and transient headache after stimulation, but usually the degree of pain is mild. The mechanism may be related to the action of variable pulse magnetic field on the scalp.

### 4.3 Results-based discussion

SRs/MAs reported methodological quality of low (50%) or very low (50%) according to AMSTAR-2. It is worth noting that 25% (2/8) of the SRs/MAs provide previous protocols, while 75% (6/8) cannot guarantee whether SRs/MAs strictly followed the research plan during the production of the research report, thus increasing the risk of bias. Additionally, 25% (2/8) SRs/MAs did not list exclusions and reasons for exclusion, which may increase selection bias. 50% (4/8) SRs/MAs did not clarify the risk of bias of individual studies, 50% (4/8) SRs/MAs did not thoroughly investigate publication bias, and 62.5% (5/8) conducted a quantitative synthesis, which made the methodological quality of the reported studies was low.

According to PRISMA, of the 27 reporting items, 16 were fully reported (100%) and 11 were omitted or underreported. Underreporting was present in the entries for protocol and registration (25%), risk of bias in individual studies (75%), synthesis of results (87.5%), risk of bias across studies (75%), additional analyses (87.5%), summary of evidence (87.5%).

Based on the results of GRADE tool evidence quality grading, 18.75% (6/32) of the results were classified as moderate evidence, while 50% (16/32) were classified as low evidence. Additionally, 31.25% (8/32) of the results were categorized as very low evidence, indicating a generally low quality of evidence. Several factors contributed to decline in quality. Firstly, the design of most studies incorporates many factors that increase bias and heterogeneity, which is the main reason for the decline in the quality of the evidence. Common factors include blinding procedures, allocation concealment, and randomization techniques. There are few recent high-quality RCT trials on rTMS for the treatment of AD, which is directly attributed to the small number of included studies, small sample sizes, inconsistent treatment selection, data extraction, and outcome labeling. Heterogeneity may also be affected by the diversity of rTMS therapy parameters and its combination with other therapies (such as cognitive training, different drugs), as well as individual differences in patients, psychological health, disease extent, and disease course factors. Secondly, the quality of evidence was also affected by publication bias (26/32), inconsistency (3/32), and imprecision (8/32) in the SRs/MAs conducted. Publication bias may arise when a small number of clinical trials with positive results are selectively published, or when there is an asymmetric funnel plot indicating potential bias in the results. Inconsistency was observed through low overlap of confidence intervals between studies and high heterogeneity in the combined results (*I*^2^ > 50%), without a reasonable explanation provided by the investigators. These factors collectively contributed to the reduced quality of evidence.

### 4.4 Implications for further study

In response to the aforementioned problems, future researchers should strive to address these limitations when conducting systematic reviews on the use of rTMS in the treatment of AD. For instance, reviewers should consider registering or publishing the protocol in advance to minimize the risk of bias and ensure the rigor of the SRs/MAs process. In terms of literature search and selection, gray literature should be taken into account, and a comprehensive list of excluded literature with explanations should be provided to ensure transparency and avoid publication bias. In cases of significant heterogeneity during data analysis, subgroup analysis should be performed. When assessing the risk of bias, it is essential to thoroughly analyze the reasons behind the bias and discuss the potential impact of publication bias. Additionally, the review should mention the funding sources, as commercially funded studies may introduce bias in favor of the funder. Researchers should adhere to the relevant guidelines such as AMSTAR-2, PRISMA, and ROBIS assessments to minimize bias and enhance the quality of the study. Moreover, when appropriate, researchers should carefully select original research and elevate the level of original research included in SRs/MAs, considering the GRADE approach.

### 4.5 Limitations

This overview has certain limitations. Differences in RCTs design and rTMS intervention details may result in higher ROB for SRs/MAs, thereby reducing the quality of the evidence and methods. The details of rTMS intervention are complex, and we cannot conduct a quantitative combined analysis of its effect size. As we have previously discussed, the effectiveness of rTMS relies on accurate positioning. If the positioning error exceeds 5 mm, rTMS may be ineffective. At present, these positions are only accurate at the anatomical, and various functional compensation and decompensation will occur after cerebral cortex injury, and the function of neural network will also change. In addition to the accurate anatomical positioning, it is worth considering whether the future can combine functional brain detection technology to achieve more accurate and individualized rTMS for better treatment efficacy and longitudinal monitoring (Rossi et al., 2021). In addition to frequency, intensity and stimulation site, continuous and intermittent rhythms may also have different effects on cognitive performance. Based on the present results, it may help in clinical decision-making for AD treatment. Because quality assessment is a subjective process and different authors may have their own judgments about each factor, the results may differ from other reviews, although our overview has been evaluated and checked by two independent reviewers.

## 5 Conclusion

Based on the evidence collected, rTMS appears to be effective and safe in improving cognitive function in AD patients, although the methodological quality of the SRs/MAs reduces the reliability of the conclusions and the overall quality is low. However, based on the available results, we still support the value of rTMS as an intervention to improve cognitive function in AD. In future studies, it is necessary to confirm the efficacy of rTMS in AD patients and provide more reliable and scientific data to contribute to evidence-based medicine.

## Data availability statement

The original contributions presented in the study are included in the article/Supplementary material, further inquiries can be directed to the corresponding authors.

## Author contributions

HX: Conceptualization, Data curation, Formal analysis, Investigation, Resources, Supervision, Validation, Visualization, Writing – original draft, Writing – review & editing. Y-xL: Investigation, Methodology, Project administration, Writing – review & editing. Y-sX: Investigation, Methodology, Writing – review & editing. W-hF: Funding acquisition, Visualization, Writing – review & editing. H-xH: Methodology, Writing – original draft, Writing – review & editing.

## References

[ref1] Aceves-Serrano L. Neva J. L. Doudet D. J. (2022). Insight into the effects of clinical repetitive transcranial magnetic stimulation on the brain from positron emission tomography and magnetic resonance imaging studies: a narrative review. Front. Neurosci. 16:787403. doi: 10.3389/fnins.2022.787403, PMID: 35264923 PMC8899094

[ref2] Ahmed M. A. Darwish E. S. Khedr E. M. El Serogy Y. M. Ali A. M. (2012). Effects of low versus high frequencies of repetitive transcranial magnetic stimulation on cognitive function and cortical excitability in Alzheimer’s dementia. J. Neurol. 259, 83–92. doi: 10.1007/s00415-011-6128-4, PMID: 21671144

[ref3] Bao Z. Bao L. Han N. Hou Y. Feng F. (2021). rTMS alleviates ad-induced cognitive impairment by inhibitng apoptosis in Samp8 mouse. Aging (Albany NY) 13, 26034–26045. doi: 10.18632/aging.203796, PMID: 34965216 PMC8751598

[ref4] Barker A. T. Jalinous R. Freeston I. L. (1985). Non-invasive magnetic stimulation of human motor cortex. Lancet 325, 1106–1107. doi: 10.1016/s0140-6736(85)92413-4 2860322

[ref5] Begemann M. J. Brand B. A. Ćurčić-Blake B. Aleman A. Sommer I. E. (2020). Efficacy of non-invasive brain stimulation on cognitive functioning in brain disorders: a meta-analysis. Psychol. Med. 50, 2465–2486. doi: 10.1017/s0033291720003670, PMID: 33070785 PMC7737055

[ref6] Birks J. S. Harvey R. J. (2018). Donepezil for dementia due to Alzheimer’s disease. Cochrane Database Syst. Rev. 6:Cd001190. doi: 10.1002/14651858.CD001190.pub3, PMID: 12917900

[ref7] Braak H. Alafuzoff I. Arzberger T. Kretzschmar H. Del Tredici K. (2006). Staging of Alzheimer disease-associated neurofibrillary pathology using paraffin sections and immunocytochemistry. Acta Neuropathol. 112, 389–404. doi: 10.1007/s00401-006-0127-z, PMID: 16906426 PMC3906709

[ref8] Chen X. Liu F. Lyu Z. Xiu H. Hou Y. Tu S. (2023). High-frequency repetitive transcranial magnetic stimulation (Hf-rTMS) impacts activities of daily living of patients with post-stroke cognitive impairment: a systematic review and meta-analysis. Neurol. Sci. 44, 2699–2713. doi: 10.1007/s10072-023-06779-9, PMID: 37012519

[ref9] Chen X. Xiu H. Hou Y. Chen X. Liu F. Tu S. (2023). High-frequency repetitive transcranial magnetic stimulation (Hf-rTMS) on overall cognition in patients with post-stroke cognitive impairment: a systematic review and meta-analysis. Am. J. Phys. Med. Rehabil. doi: 10.1097/phm.0000000000002377, PMID: 38113027

[ref10] Cheng J. Wu W. (2016). Meta-analysis of the effect of high-frequency repetitive transcranial magnetic stimulation on Alzheimer’s disease. Chin J Practic Med 32, 2539–2543. doi: 10.3969/j.issn.1006-5725.2016.15.037

[ref11] Dong X. Yan L. Huang L. Guan X. Dong C. Tao H. . (2018). Repetitive transcranial magnetic stimulation for the treatment of Alzheimer’s disease: a systematic review and meta-analysis of randomized controlled trials. PLoS One 13:e0205704. doi: 10.1371/journal.pone.0205704, PMID: 30312319 PMC6185837

[ref12] Dressler D. Voth E. Feldmann M. Benecke R. (1990). Safety aspects of transcranial brain stimulation in man tested by single photon emission-computed tomography. Neurosci. Lett. 119, 153–155. doi: 10.1016/0304-3940(90)90821-p, PMID: 2280887

[ref13] Jannati A. Oberman L. M. Rotenberg A. Pascual-Leone A. (2023). Assessing the mechanisms of brain plasticity by transcranial magnetic stimulation. Neuropsychopharmacology 48, 191–208. doi: 10.1038/s41386-022-01453-8, PMID: 36198876 PMC9700722

[ref14] Kaur D. Sharma V. Deshmukh R. (2019). Activation of microglia and astrocytes: a roadway to neuroinflammation and Alzheimer’s disease. Inflammopharmacology 27, 663–677. doi: 10.1007/s10787-019-00580-x, PMID: 30874945

[ref15] Kim T. D. Hong G. Kim J. Yoon S. (2019). Cognitive enhancement in neurological and psychiatric disorders using transcranial magnetic stimulation (Tms): a review of modalities, potential mechanisms and future implications. Exp Neurobiol 28, 1–16. doi: 10.5607/en.2019.28.1.1, PMID: 30853820 PMC6401552

[ref16] Lefaucheur J. P. Aleman A. Baeken C. Benninger D. H. Brunelin J. Di Lazzaro V. . (2020). Evidence-based guidelines on the therapeutic use of repetitive transcranial magnetic stimulation (rTMS): an update (2014–2018). Clin. Neurophysiol. 131, 474–528. doi: 10.1016/j.clinph.2019.11.002, PMID: 31901449

[ref17] Leng F. Edison P. (2021). Neuroinflammation and microglial activation in Alzheimer disease: where do we go from here? Nat. Rev. Neurol. 17, 157–172. doi: 10.1038/s41582-020-00435-y, PMID: 33318676

[ref18] Li Y. Li L. Pan W. (2019). Repetitive transcranial magnetic stimulation (rTMS) modulates hippocampal structural synaptic plasticity in rats. Physiol. Res. 68, 99–105. doi: 10.33549/physiolres.933772 30433805

[ref19] Liang B. Wang X. W. Zhang H. Wang Y. Liang T. Tang J. T. (2017). Meta-analysis of transcranial magnetic stimulation in patients with Alzheimer’s disease. Sci Technol Herald 35, 95–99.

[ref20] Liao X. Li G. Wang A. Liu T. Feng S. Guo Z. . (2015). Repetitive transcranial magnetic stimulation as an alternative therapy for cognitive impairment in Alzheimer’s disease: a meta-analysis. J. Alzheimers Dis. 48, 463–472. doi: 10.3233/jad-150346, PMID: 26402010

[ref21] Lin Y. Jiang W. J. Shan P. Y. Lu M. Wang T. Li R. H. . (2019). The role of repetitive transcranial magnetic stimulation (rTMS) in the treatment of cognitive impairment in patients with Alzheimer’s disease: a systematic review and meta-analysis. J. Neurol. Sci. 398, 184–191. doi: 10.1016/j.jns.2019.01.038, PMID: 30735817

[ref22] Marucci G. Buccioni M. Ben D. D. Lambertucci C. Volpini R. Amenta F. (2021). Efficacy of acetylcholinesterase inhibitors in Alzheimer’s disease. Neuropharmacology 190:108352. doi: 10.1016/j.neuropharm.2020.108352 33035532

[ref23] Nguyen J. P. Suarez A. Le Saout E. Meignier M. Nizard J. Lefaucheur J. P. (2018). Combining cognitive training and multi-site rTMS to improve cognitive functions in Alzheimer’s disease. Brain Stimul. 11, 651–652. doi: 10.1016/j.brs.2018.02.013, PMID: 29519726

[ref24] O’Reardon J. P. Solvason H. B. Janicak P. G. Sampson S. Isenberg K. E. Nahas Z. . (2007). Efficacy and safety of transcranial magnetic stimulation in the acute treatment of major depression: a multisite randomized controlled trial. Biol. Psychiatry 62, 1208–1216. doi: 10.1016/j.biopsych.2007.01.018, PMID: 17573044

[ref25] Page M. J. Moher D. Bossuyt P. M. Boutron I. Hoffmann T. C. Mulrow C. D. . (2021). Prisma 2020 explanation and elaboration: updated guidance and exemplars for reporting systematic reviews. BMJ 372:n160. doi: 10.1136/bmj.n160, PMID: 33781993 PMC8005925

[ref26] Pardo-Moreno T. González-Acedo A. Rivas-Domínguez A. García-Morales V. García-Cozar F. J. Ramos-Rodríguez J. J. . (2022). Therapeutic approach to Alzheimer’s disease: current treatments and new perspectives. Pharmaceutics 14:61117. doi: 10.3390/pharmaceutics14061117, PMID: 35745693 PMC9228613

[ref27] Rossi S. Antal A. Bestmann S. Bikson M. Brewer C. Brockmöller J. . (2021). Safety and recommendations for TMS use in healthy subjects and patient populations, with updates on training, ethical and regulatory issues: expert guidelines. Clin. Neurophysiol. 132, 269–306. doi: 10.1016/j.clinph.2020.10.003, PMID: 33243615 PMC9094636

[ref28] Schünemann H. J. Oxman A. D. Brozek J. Glasziou P. Jaeschke R. Vist G. E. . (2008). Grading quality of evidence and strength of recommendations for diagnostic tests and strategies. BMJ 336, 1106–1110. doi: 10.1136/bmj.39500.677199.AE, PMID: 18483053 PMC2386626

[ref29] Shea B. J. Reeves B. C. Wells G. Thuku M. Hamel C. Moran J. . (2017). Amstar 2: a critical appraisal tool for systematic reviews that include randomised or non-randomised studies of healthcare interventions, or both. BMJ 358:j4008. doi: 10.1136/bmj.j4008, PMID: 28935701 PMC5833365

[ref30] Silva M. V. F. Loures C. M. G. Alves L. C. V. de Souza L. C. Borges K. B. G. Carvalho M. D. G. (2019). Alzheimer’s disease: risk factors and potentially protective measures. J. Biomed. Sci. 26:33. doi: 10.1186/s12929-019-0524-y, PMID: 31072403 PMC6507104

[ref31] Song P. Li S. Wang S. Wei H. Lin H. Wang Y. (2020). Repetitive transcranial magnetic stimulation of the cerebellum improves ataxia and Cerebello-Fronto plasticity in multiple system atrophy: a randomized, double-blind, sham-controlled and Tms-Eeg study. Aging (Albany NY) 12, 20611–20622. doi: 10.18632/aging.103946, PMID: 33085647 PMC7655163

[ref32] Turriziani P. Smirni D. Mangano G. R. Zappalà G. Giustiniani A. Cipolotti L. . (2019). Low-frequency repetitive transcranial magnetic stimulation of the right dorsolateral prefrontal cortex enhances recognition memory in Alzheimer’s disease. J. Alzheimers Dis. 72, 613–622. doi: 10.3233/jad-190888, PMID: 31609693

[ref33] van der Kant R. Goldstein L. S. B. Ossenkoppele R. (2020). Amyloid-Β-independent regulators of tau pathology in Alzheimer disease. Nat. Rev. Neurosci. 21, 21–35. doi: 10.1038/s41583-019-0240-3, PMID: 31780819

[ref34] Wang X. Mao Z. Ling Z. Yu X. (2020). Repetitive transcranial magnetic stimulation for cognitive impairment in Alzheimer’s disease: a meta-analysis of randomized controlled trials. J. Neurol. 267, 791–801. doi: 10.1007/s00415-019-09644-y 31760522

[ref35] Wang R. Reddy P. H. (2017). Role of glutamate and NMDA receptors in Alzheimer’s disease. J. Alzheimers Dis. 57, 1041–1048. doi: 10.3233/jad-160763, PMID: 27662322 PMC5791143

[ref36] Watermeyer T. Calia C. (2019). Neuropsychological assessment in preclinical and prodromal Alzheimer disease: a global perspective. J. Glob. Health 9:10317. doi: 10.7189/jogh.09.010317, PMID: 31073397 PMC6486120

[ref37] Whiting P. Savović J. Higgins J. P. Caldwell D. M. Reeves B. C. Shea B. . (2016). Robis: a new tool to assess risk of bias in systematic reviews was developed. J. Clin. Epidemiol. 69, 225–234. doi: 10.1016/j.jclinepi.2015.06.005, PMID: 26092286 PMC4687950

[ref38] Xiu H. Liu F. Hou Y. Chen X. Tu S. (2024). High-frequency repetitive transcranial magnetic stimulation (Hf-rTMS) on global cognitive function of elderly in mild to moderate Alzheimer’s disease: a systematic review and meta-analysis. Neurol. Sci. 45, 13–25. doi: 10.1007/s10072-023-07072-5, PMID: 37749398

[ref39] XXX (2023). 2023 Alzheimer’s disease facts and figures. Alzheimers Dement. 19, 1598–1695. doi: 10.1002/alz.13016, PMID: 36918389

[ref40] Zhang T. Sui Y. Lu Q. Xu X. Zhu Y. Dai W. . (2022). Effects of rTMS treatment on global cognitive function in Alzheimer’s disease: a systematic review and meta-analysis. Front. Aging Neurosci. 14:984708. doi: 10.3389/fnagi.2022.984708, PMID: 36158564 PMC9492846

