# Supplementary material for: Repetitive transcranial magnetic stimulation for Alzheimer’s disease: an overview of systematic reviews and meta-analysis
Source: Front Aging Neurosci. 2024 Mar 20;16:1383278. doi: 10.3389/fnagi.2024.1383278 (PMC10987751; doi:10.3389/fnagi.2024.1383278)
Supplement: Supplementary file 1 [file Data_Sheet_1.docx]

**Supplementary material**

***Supplementary Table 1:*** The search strategy for each database

***Pubmed***

#1 "Transcranial Magnetic Stimulation"[MeSH Terms] OR "Transcranial Direct Current Stimulation"[MeSH Terms]

#2 "noninvasive brain stimulation"[Title/Abstract] OR "non invasive brain stimulation"[Title/Abstract] OR "brain stimulation"[Title/Abstract] OR "tDCS"[Title/Abstract] OR "magnetic stimulations"[Title/Abstract] OR "magnetic stimulation"[Title/Abstract] OR "transcranial magnetic stimulation"[Title/Abstract] OR "repetitive transcranial magnetic stimulation"[Title/Abstract]

#3 #1 OR #2

#4 "Alzheimer Disease"[Mesh]

#5 Alzheimer Disease[Title/Abstract] OR Alzheimer’s Disease[Title/Abstract] OR Alzheimer’s disease[Title/Abstract] OR Alzheimer Dementia[Title/Abstract] OR Alzheimer Dementias[Title/Abstract] OR Alzheimer Type Dementia[Title/Abstract] OR Alzheimer’s Diseases[Title/Abstract] OR Early Onset Alzheimer Disease[Title/Abstract] OR Late Onset Alzheimer Disease[Title/Abstract] OR Familial Alzheimer Diseases[Title/Abstract] OR Familial Alzheimer Disease[Title/Abstract]

#6 #4 OR #5

#7 " meta analysis "[Mesh]

#8 "meta analysis"[Title/Abstract] OR "meta analysis"[Title/Abstract] OR "systematic review"[Title/Abstract] OR "systematic assessment"[Title/Abstract] OR "system evaluation"[Title/Abstract] OR "systematic evaluation"[Title/Abstract] OR "meta analysis as topic"[MeSH Major Topic]

#9 #7 OR #8

#10 #9 OR #6 OR #3

***Embase***

#1 ‘meta analysis’/ exp OR ‘meta analysis (topic)’/ exp

#2 ‘meta analysis’: ti,ab OR ‘meta analyses’: ti,ab OR ‘meta-analysis’: ti,ab OR ‘meta-analyses’: ti,ab OR meta analysis:ti,ab OR meta analysis: ti,ab OR ‘met-analysis’: ti, ab OR meta analyses: ti, ab OR metanalyses: ti,ab OR ‘clinical trial overview’: ti, ab OR ‘clinical trial overviews’: ti,ab

#3 ‘systematic review’/ exp OR ‘systematic review (topic)’/ exp

#4 ‘systematic review’: ti,ab OR ‘systematic reviews’: ti, ab

#5 #1 OR #2 OR #3 OR #4

#6 ‘repetitive transcranial magnetic stimulation’: ti,ab OR ‘transcranial magnetic stimulation’ : ti,ab OR ‘noninvasive brain stimulation’ : ti,ab

#7‘repetitive transcranial magnetic stimulation’/ exp OR ‘transcranial magnetic stimulation’ / exp OR ‘noninvasive brain stimulation’ / exp

#8 #6 OR #7

#9 ‘Alzheimer Disease’/ exp OR ‘Alzheimer’s Disease’ / exp OR ‘Alzheimer Dementia’ / exp

#10‘Alzheimer Disease’ : ti,ab OR ‘Alzheimer’s Disease’ : ti,ab OR ‘Alzheimer Dementia’ : ti,ab

#11 #9 OR #10

#12 #5 AND #8 AND #11

***Web of Science***

#1 TS =“meta analysis” OR TS =“meta analyses” OR TS =“meta-analysis” OR TS =“meta-analyses” OR TS = “meta-Analysis” OR TS =“metanalysis”OR TS =“ metaanalyses”OR TS =“systematic review” OR TS =“systematic reviews” OR TS =“clinical trial overview” OR TS =“clinical trial overviews”

#2 TS =“repetitive transcranial magnetic stimulation” OR TS =“transcranial magnetic stimulation” OR TS =“noninvasive brain stimulation”

#3 TS =“Alzheimer Disease” OR TS =“Alzheimer’s Disease” OR TS =“Alzheimer Dementia”

#4 #1 AND #2 AND #3

***The Cochrane Library***

#1 MeSH descriptor: [Alzheimer’s Disease] explode all trees

#2 (Alzheimer’s Disease):ab,ti,kw OR (Alzheimer Disease):ab,ti,kw

#3 #1OR#2

#4 (meta analysis):ab,ti,kw OR (meta analyses):ab,ti,kw OR (meta-analysis):ab,ti,kw OR (meta-analyses):ab,ti,kw OR (meta-Analysis):ab,ti,kw OR (metanalysis):ab,ti,kw OR (metaanalyses):ab,ti,kw OR (systematic review):ab,ti,kw OR (systematic reviews):ab,ti,kw OR (clinical trial overview):ab,ti,kw OR (clinical trial overviews):ab,ti,kw

#5 (repetitive transcranial magnetic stimulation):ab,ti,kw OR (transcranial magnetic stimulation):ab,ti,kw OR (noninvasive brain stimulation):ab,ti,kw

#6 #3 AND #4 AND #5

***China National Knowledge Infrastructure***

( ( (主题=系统评价 或者 题名=系统评价 或者 v_subject=中英文扩展(系统评价) 或者 title=中英文扩展(系统评价)) 或者 (主题=meta分析 或者 题名=meta分析 或者 v_subject=中英文扩展(meta分析) 或者 title=中英文扩展(meta分析)) ) 或者 ( (主题=荟萃分析 或者 题名=荟萃分析 或者 v_subject=中英文扩展(荟萃分析) 或者 title=中英文扩展(荟萃分析)) 或者 (主题=元分析 或者 题名=元分析 或者 v_subject=中英文扩展(元分析) 或者 title=中英文扩展(元分析)) ) ) 并且 ( ( (主题=重复经颅磁刺激 或者 题名=重复经颅磁刺激 或者 v_subject=中英文扩展(重复经颅磁刺激) 或者 title=中英文扩展(重复经颅磁刺激)) 或者 (主题=经颅磁刺激 或者 题名=经颅磁刺激 或者 v_subject=中英文扩展(经颅磁刺激) 或者 title=中英文扩展(经颅磁刺激)) ) 或者 ( (主题=无创性脑刺激 或者 题名=无创性脑刺激 或者 v_subject=中英文扩展(无创性脑刺激) 或者 title=中英文扩展(无创性脑刺激)) ) ) 并且 ( ( (主题=阿尔茨海默病 或者 题名=阿尔茨海默病 或者 v_subject=中英文扩展(阿尔茨海默病) 或者 title=中英文扩展(阿尔茨海默病))

***Wanfang Database***

检索表达式（中英文扩展&主题词扩展）： 主题:(系统评价+系统综述+meta分析+荟萃分析+元分析)*主题:(经颅磁刺激+重复经颅磁刺激+无创性脑刺激)*主题:(阿尔茨海默病+阿尔茨海默症+阿尔茨海默患者+阿尔茨海默)

***Chongqing VIP***

(U=阿尔茨海默病 OR U=阿尔茨海默患者 OR U=阿尔茨海默症) AND (U=重复经颅磁刺激 OR U=经颅磁刺激 OR U=无创性脑刺激 OR U=非侵入性脑刺激) AND (U=荟萃分析 OR U=系统综述 OR U=系统评价 OR U=Meta分析)

***Sino-Med***

1 "阿尔茨海默病"[不加权:扩展]

2 "经颅磁刺激"[不加权:扩展]

3 "重复经颅磁刺激"[不加权:扩展]

4 "非侵入性脑刺激"[不加权:扩展]

5 "经颅磁刺激"[常用字段:智能] OR "重复经颅磁刺激"[常用字段:智能] OR "非侵入性脑刺激"[常用字段:智能] OR "无创性脑刺激"[常用字段:智能]

6 (#2) OR (#3) OR (#4) OR (#5)

7 "Meta分析"[不加权:扩展]

8 "Meta分析"[常用字段:智能] OR "系统评价"[常用字段:智能] OR "荟萃分析"[常用字段:智能] OR "系统综述"[常用字段:智能]

9 (#7) OR (#8)

10 (#1) AND (#6) AND (#9)
